# Supplementary material for: Auxiliary subunits reshape structural asymmetry and functional plasticity in heterotetrameric GluA1/A2 AMPA receptor core
Source: Nat Commun. 2026 Mar 28;17:4191. doi: 10.1038/s41467-026-71063-1 (PMC13153358; doi:10.1038/s41467-026-71063-1)
Supplement: Supplementary file 1 — Supplementary Information [file 41467_2026_71063_MOESM1_ESM.pdf]

Supplementary Information

Auxiliary subunits reshape structural asymmetry and functional plasticity in heterotetrameric GluA1/A2 AMPA receptor core

Laura Y. Yen[1,2], Thomas P. Newton[1,3], Maria V. Yelshanskaya[1], Muhammed Aktolun[4], Shanti Pal Gangwar[1], Rasmus P. Clausen[5], Maria G. Kurnikova[4], and Alexander I. Sobolevsky[1#]

[1] Department of Biochemistry and Molecular Biophysics, Columbia University, 650 West 168th Street, New York, NY 10032, USA

[2] Cellular and Molecular Physiology and Biophysics Graduate Program, Columbia University Irving Medical Center, 630 West 168th Street, New York, NY 10032, USA

[3] Cellular, Molecular, and Biomedical Studies Umbrella Program, Columbia University Irving Medical Center, 630 West 168th Street, New York, NY 10032, USA.

[4] Department of Chemistry, Carnegie Mellon University, Pittsburgh, PA 15213, USA.

[5] Department of Drug Design and Pharmacology, University of Copenhagen, Universitetsparken 2, 2100 Copenhagen, Denmark.

[#] Correspondence and requests for materials should be addressed to A.I.S.
(Email: as4005@cumc.columbia.edu; Tel: 212-305-4249)

This PDF file includes:

Supplementary Figures 1-9

Supplementary Table 1

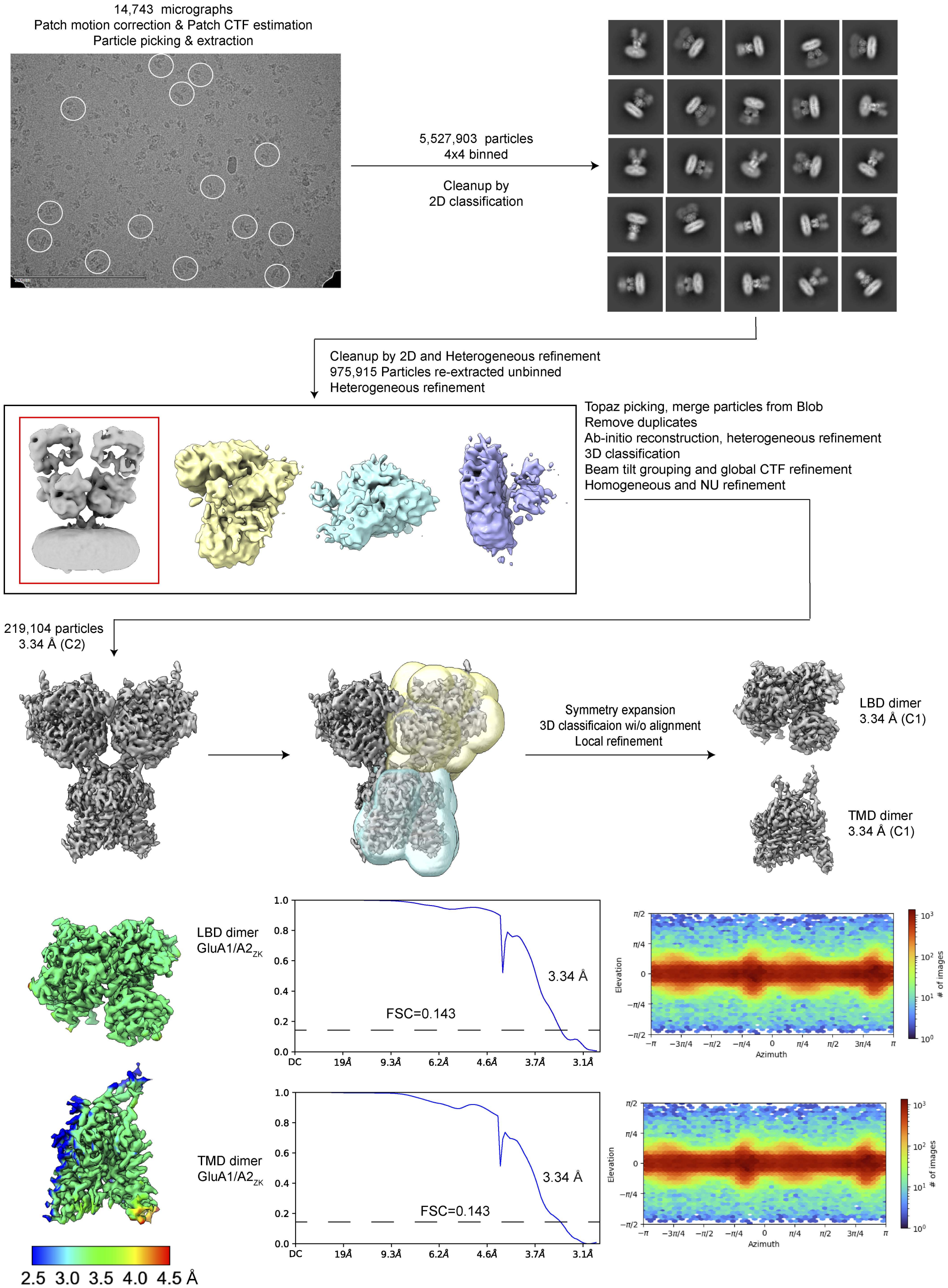

Supplementary Figure 1. Overview of cryo-EM for GluA1/A2$_{ZK}$. At the top is a flowchart outlining cryo-EM image acquisition and processing workflow performed to obtain the structure of GluA1/A2$_{ZK}$. Briefly, raw data (movies) were collected using a 300-kV Krios transmission electron microscope. Movies were motion-corrected and subjected to CTF estimation. Particles were selected by Template-based and Topaz pickers and subjected to 2D and 3D classification. A subset of the best particles yielded a 3.4-Å resolution consensus reconstruction of the LBD-TMD. We then performed symmetry expansion (C2) with further 3D classification. Finally, local refinement of the LBD dimer (A/D) and TMD dimer (A/B) improved the final resolution to 3.34 Å, as displayed by cryo-EM maps colored according to the local resolution estimation in cryoSPARC (lower left). Blue pattern reflects the higher resolution at the TMD core. FSC curves calculated between half maps, with the overall resolution estimated using the FSC = 0.143 criterion and angular distribution of particles calculated using the 3D refinement reconstruction algorithm in cryoSPARC are shown in the lower middle and lower right.

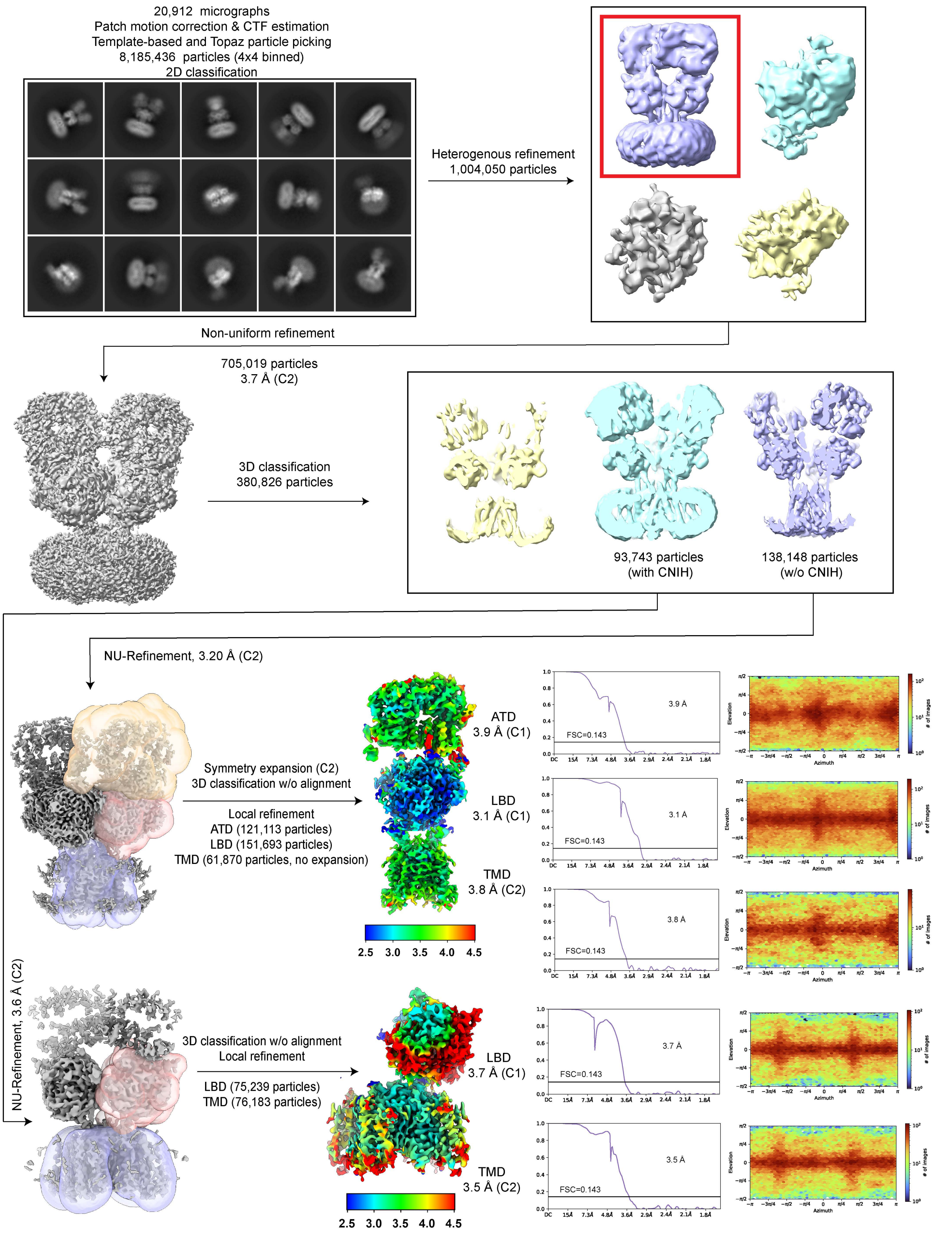

Supplementary Figure 2. Overview of cryo-EM for GluA1/A2 in the presence of Glu and RR2b. At the top is a flowchart that outlines cryo-EM image acquisition and processing workflow performed to obtain the structures of GluA1/A2$_{Glu+RR2b}$ and GluA1/A2-CNIH$_{Glu+RR2b}$. At the bottom, there are cryo-EM maps colored according to the local resolution estimation in cryoSPARC (left), FSC curves calculated between half maps, with the overall resolution estimated using the FSC = 0.143 criterion (middle) and angular distribution of particles calculated using the 3D refinement reconstruction algorithm in cryoSPARC (right).

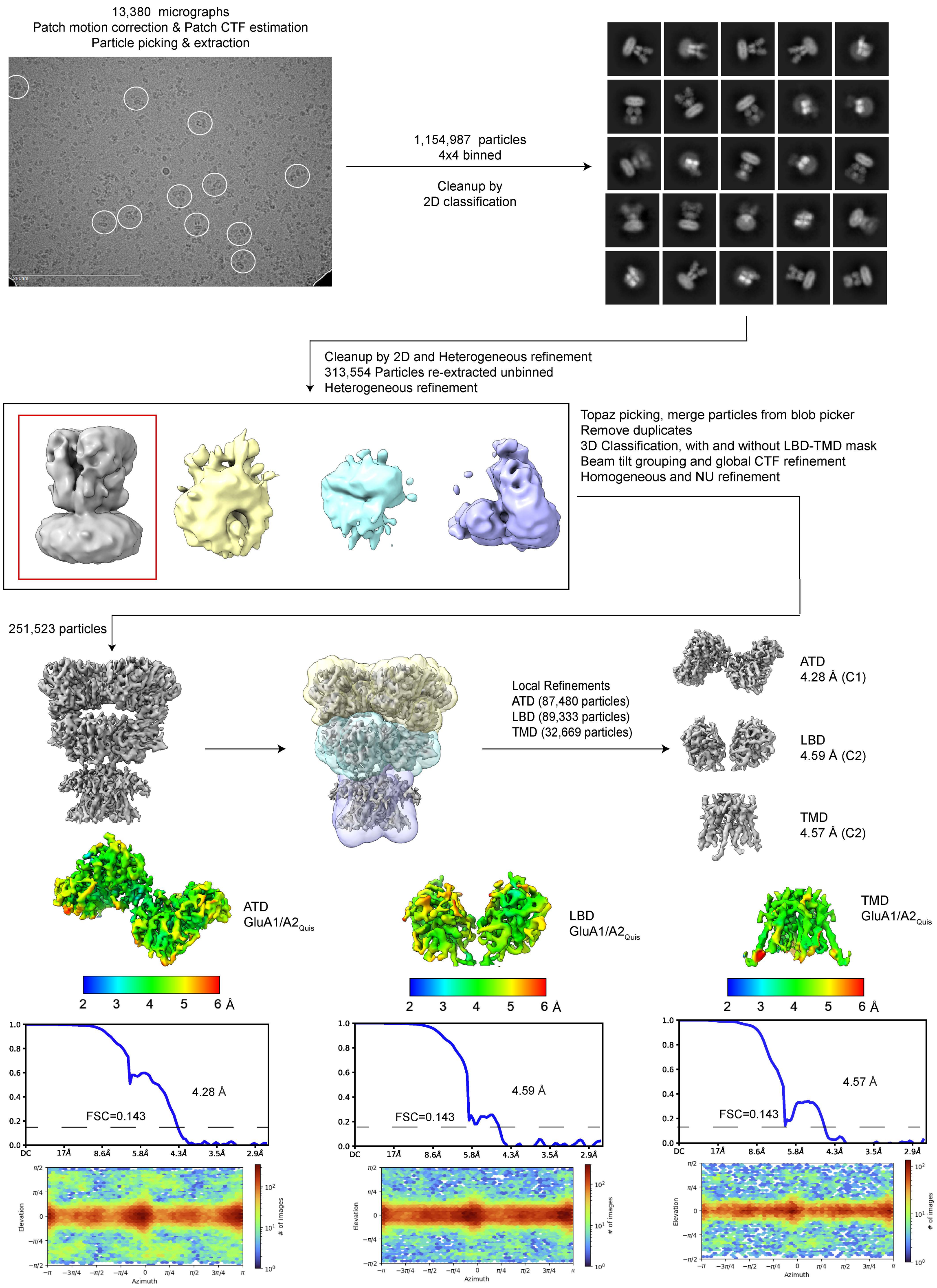

Supplementary Figure 3. Overview of cryo-EM for GluA1/A2$_{Quis}$. At the top is a flowchart that outlines cryo-EM image acquisition and processing workflow performed to obtain the desensitized-state GluA1/A2$_{Quis}$ structure, with a representative micrograph showing example particles circled in white and 2D class averages. Shown at the bottom are cryo-EM maps colored according to the local resolution estimation in cryoSPARC, FSC curves calculated between half maps, with the overall resolution estimated using the FSC = 0.143 criterion and angular distribution of particles calculated using the 3D refinement reconstruction algorithm in cryoSPARC.

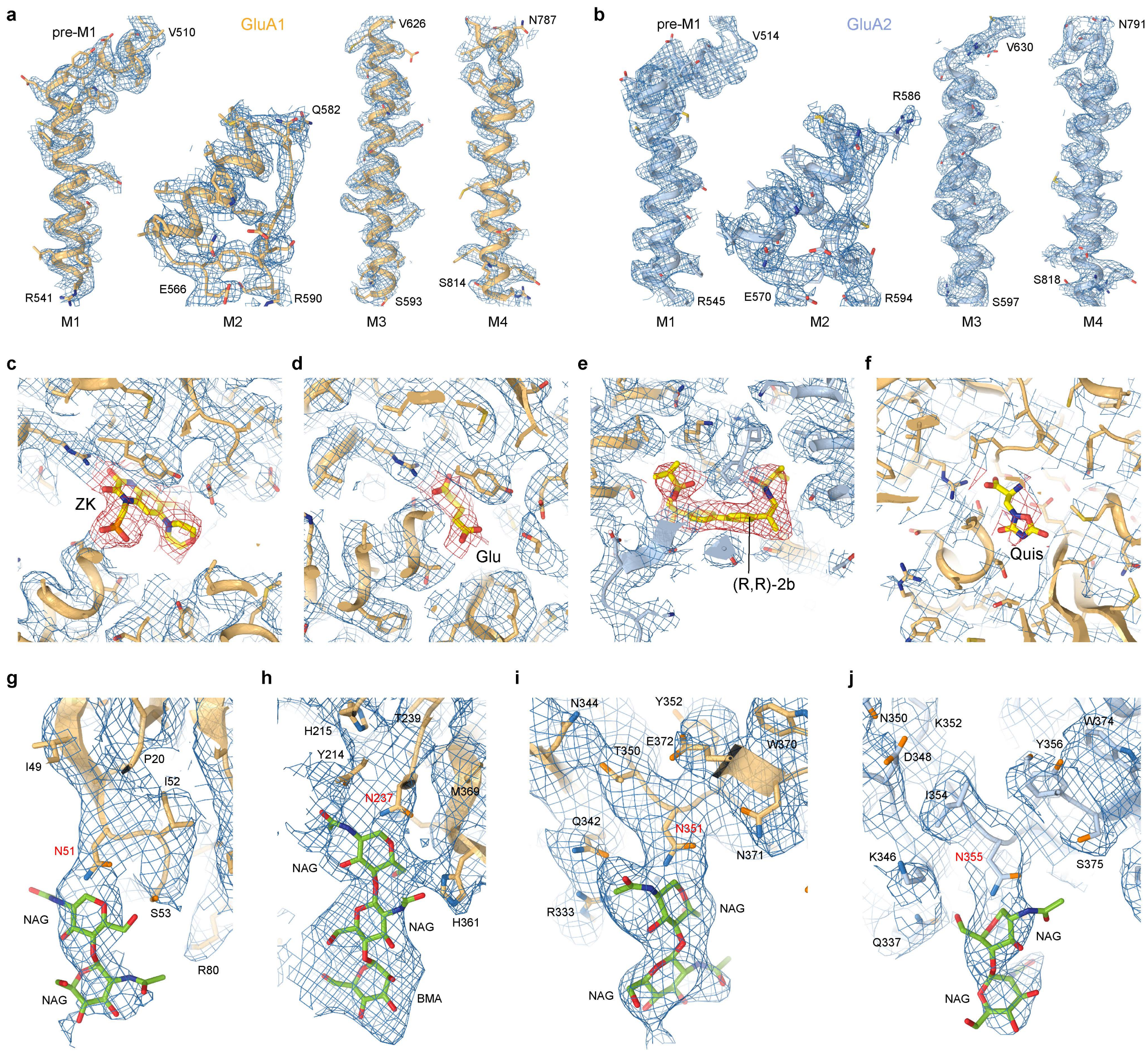

Supplementary Figure 4. Cryo-EM density for AMPARs. a-b, Fragments of cryo-EM density (blue mesh) for the TMD segments of GluA1 (**a**) and GluA2 (**b**). **c-f**, Binding pockets of ZK (**c**), Glu (**d**), (R,R)-2b (**e**), and Quis (**f**), with the small molecules (yellow) and surrounding residues (orange for GluA1 and blue for GluA2) shown in sticks and the corresponding cryo-EM density as red and blue mesh, respectively. **g-j**, Unique N-linked glycosylation sites in GluA1 (**g-i**) and GluA2 (**j**), which unambiguously determine the identity of AMPAR subunits and their order in the tetramer, with the glycosylated asparagines (red labels) N51 (**g**), N237 (**h**), N351 (**i**) and N355 (**j**), and NAG and BMA carbohydrates (green) shown in sticks and the corresponding cryo-EM density shown as blue mesh.

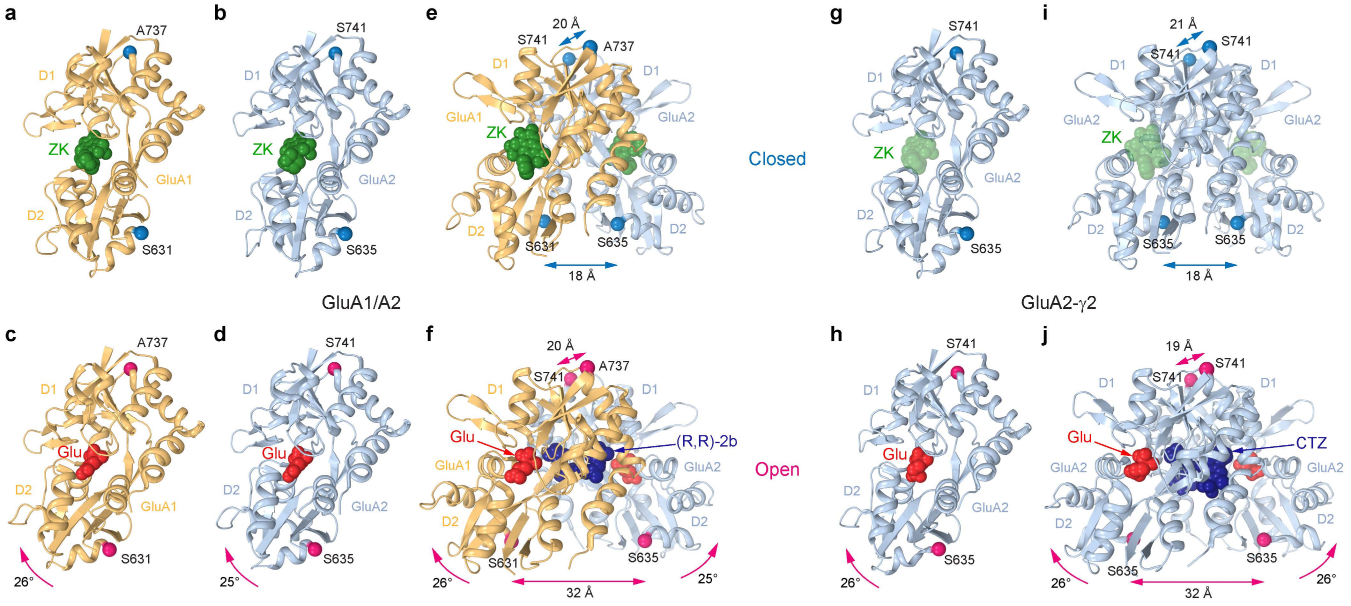

Supplementary Figure 5. Activation at the level of LBD monomers and dimers. a-d, LBDs of GluA1 (**a,c**) and GluA2 (**b,d**) subunits in the closed-state GluA1/A2$_{ZK}$ (**a-b**) and open-state GluA1/A2$_{Glu+RR2b}$ (**c-d**) structures, with molecules of ZK and Glu shown as green and red space-filling models, GluA1 (A/C) and GluA2 (B/D) subunits colored light orange and blue and LBD upper (D1) and lower (D2) lobes labeled. 26° and 25° rotations of the D2 lobes toward D1 in GluA1 (**c**) and GluA2 (**d**) of GluA1/A2$_{Glu+RR2b}$ compared to GluA1/A2$_{ZK}$ are illustrated by the pink arrows. **e-f**, Structures of LBD dimers in GluA1/A2$_{ZK}$ (**e**) and GluA1/A2$_{Glu+RR2b}$ (**f**), with the molecule of (R,R)-2b shown as a dark blue space-filling model and two-sided arrows indicating distances between the upper or lower LBD lobes. Closure of the individual LBD clamshells in GluA1/A2$_{Glu+RR2b}$ is indicated by one-sided pink arrows. **g-h**, LBDs of GluA2 in the closed-state GluA2-γ2$_{ZK}$ (**g**, PDB ID: 5KK2) and open-state GluA2-γ2$_{Glu+CTZ}$ (**h**, PDB ID: 5WEO) structures. Approximate position of the ZK molecule that was not modeled in GluA2-γ2$_{ZK}$ is shown by the transparent space-filling model (green). **i-j**, Structures of LBD dimers in GluA2-γ2$_{ZK}$ (**i**) and GluA2-γ2$_{Glu+CTZ}$ (**j**), with the molecules of CTZ shown as dark blue space-filling models. Note, the behavior during AMPAR activation at both individual LBD clamshell (**a-d** and **g-h**) and LBD dimer (**e-f** and **i-j**) levels is similar between heteromeric GluA1/A2 (**a-f**) and homomeric GluA2 (**g-j**) receptors.

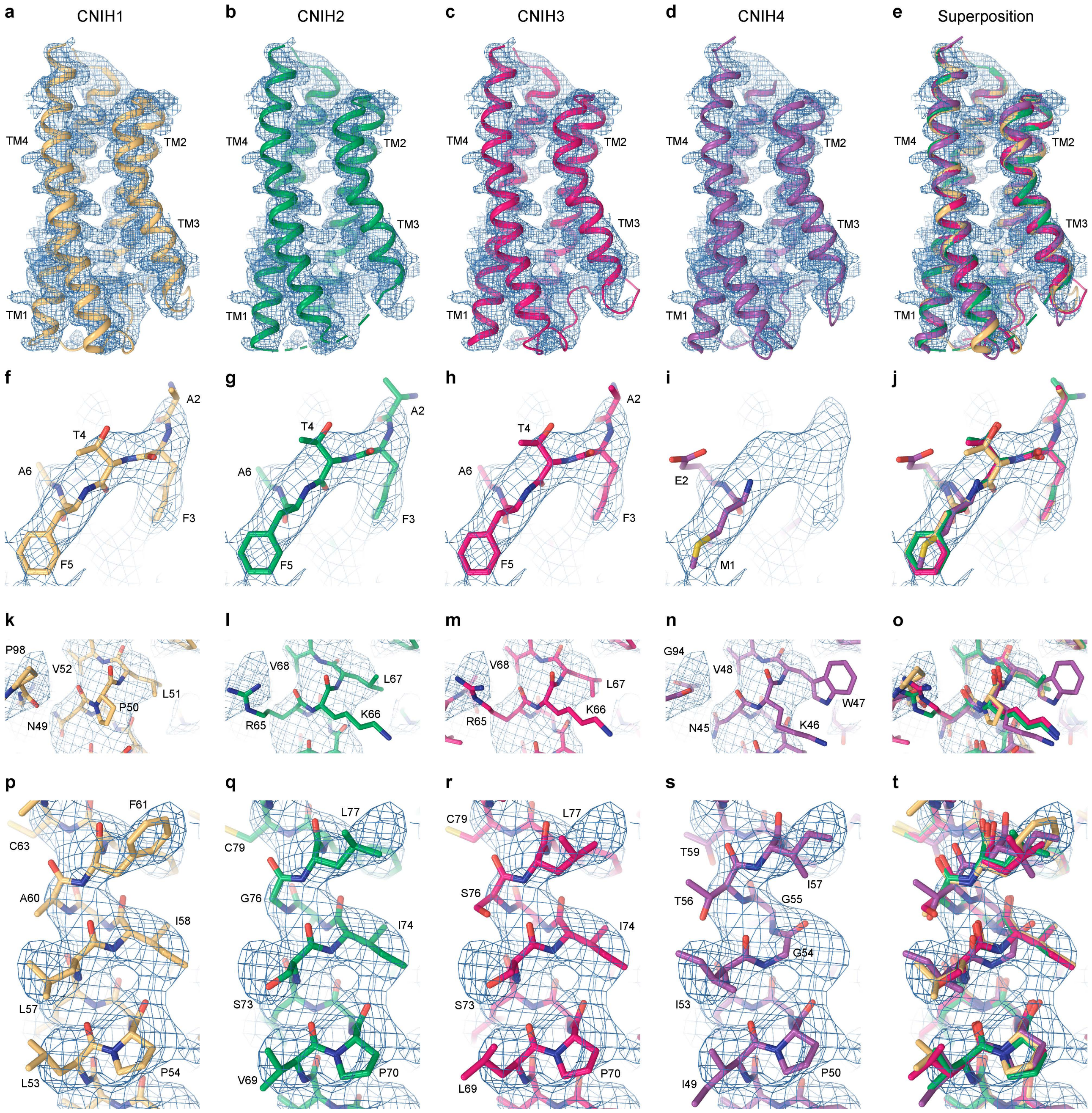

Supplementary Figure 6. **Cryo-EM density for CNIHs. a-e**, Cryo-EM map for the auxiliary subunit in GluA1/A2-CNIH$_{Glu+RR2b}$ (blue mesh) with fitted models (cartoon) of human CNIH1 (**a**), CNIH2 (**b**), CNIH3 (**c**), CNIH4 (**d**) and their superposition (**e**). Transmembrane helices (TM1–TM4) are labeled. **f-t**, Close-up views of the N-terminus (**f-j**), as well as N-terminal (**k-o**) and central (**p-t**) parts of TM2, with the protein shown in sticks, illustrating different fits of the models into the cryo-EM map.

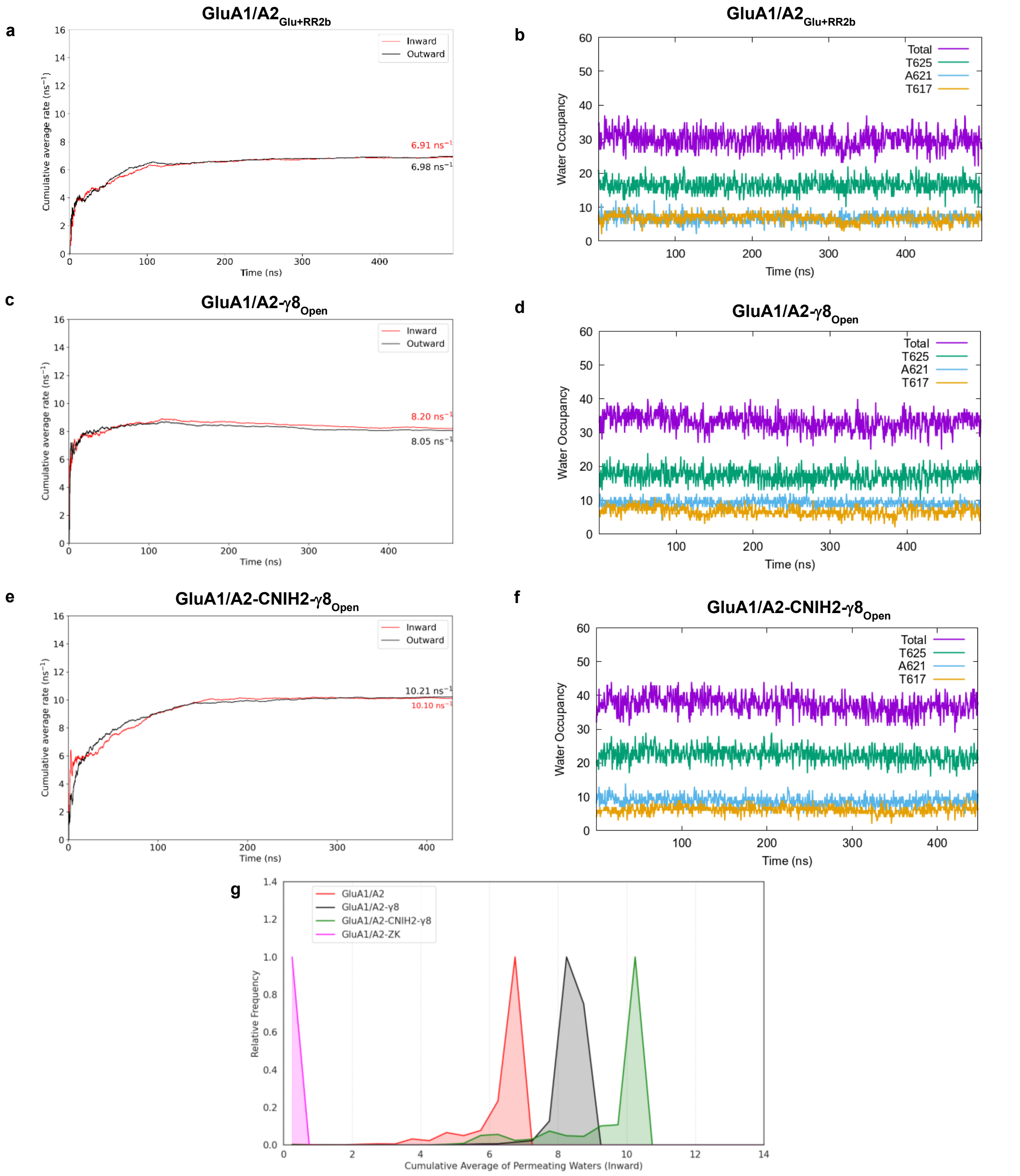

Supplementary Figure 7. Water conductance rates and gate water occupancies. a,c,e, Cumulative average permeation rate of water molecules during the MD simulations of GluA1/A2$_{Glu+RR2b}$ (**a**), GluA1/A2-γ8$_{Open}$ (PDB ID: 7QHB, **c**) and GluA1/A2-CNIH2-γ8$_{Open}$ (PDB ID: 7OCF, **e**). Inward (to intracellular) and outward (to extracellular) water permeation rates per nanosecond are shown as red and black lines, respectively. Corresponding rates for inward and outward conductances are shown next to each line. Water permeation was calculated as the number of water molecules crossing the space between levels T625 and T617 Cα atoms per ns. Cumulative average was calculated by integrating the mean permeation count over the duration of the simulation**. b,d,f,** Occupancies of water molecules at the gate residues T617 (yellow), A621 (blue), T625 (green) and total number of waters at these residue levels (purple) during the simulations of GluA1/A2$_{Glu+RR2b}$ (**b**), GluA1/A2-γ8$_{Open}$ (PDB ID: 7QHB, **d**) and GluA1/A2-CNIH2-γ8$_{Open}$ (PDB ID: 7OCF, **f**). **g**, Normalized histograms showing frequencies the inward cumulative average permeation rates for the equilibration simulations of GluA1/A2$_{Glu+RR2b}$ (red), GluA1/A2-γ8$_{Open}$ (PDB ID: 7QHB, black) and GluA1/A2-CNIH2-γ8$_{Open}$ (PDB ID: 7OCF, green), and production simulation of closed GluA1/A2$_{ZK}$ (magenta).

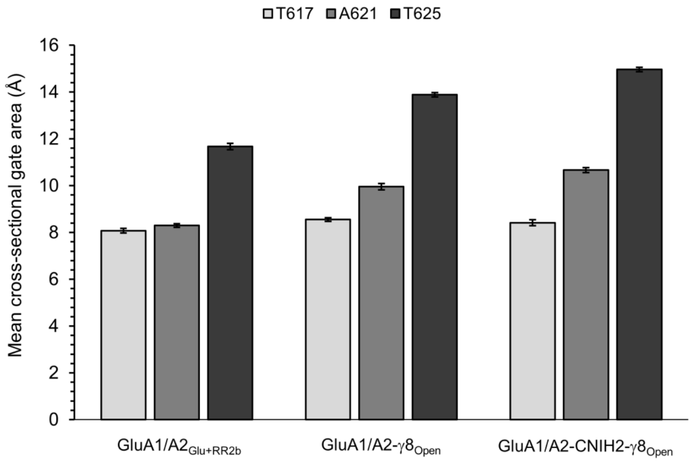

Supplementary Figure 8. Mean cross-sectional gate areas. Areas at the gate residue levels T617 (light gray), A621 (medium gray) and T625 (dark gray) during the backbone-restrained simulations of GluA1/A2$_{Glu+RR2b}$, GluA1/A2-γ8$_{Open}$ (PDB ID: 7QHB) and GluA1/A2-CNIH2-γ8$_{Open}$ (PDB ID: 7OCF). Areas were calculated using the Cα atoms of each residue from the four subunits and reported in angstrom (Å) as the square root of actual value. Error bars represent the standard deviation of the gate area values within the trajectories.

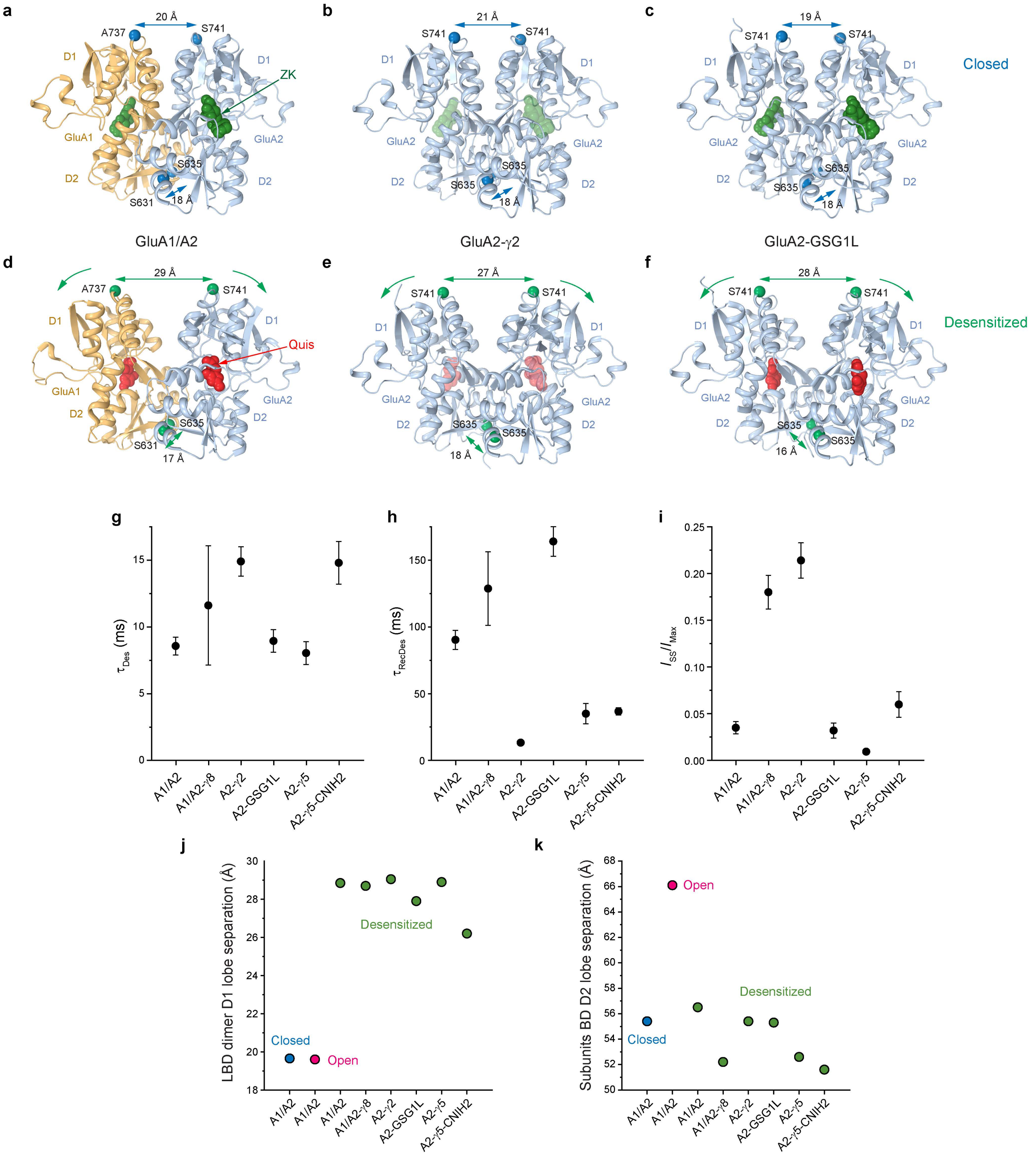

Supplementary Figure 9. Structural and functional characteristics of desensitization. a-f, LBD dimer structures for GluA1/A2 (**a,d**), GluA2-γ2 (**b**, PDB ID: 5KK2; **e**, PDB ID: 5VOV) and GluA2-GSG1L (**c**, PDB ID: 5VHY; **f**, PDB ID: 5VHZ) in the closed (**a-c**) and desensitized (**d-f**) states, with GluA1 and GluA2 subunits colored light orange and blue, respectively. Molecules of ZK and Quis are shown as green and red space-filling models. Transparency of the space-filling model of ZK in GluA2-$\gamma2_{ZK}$ indicates that the inhibitor molecule was not modeled in the original structure and its placement is approximate. Rotation of the LBD D1 lobes accompanying D1-D1 interface rupture during desensitization compared to the closed state are indicated by one-sided green arrows. Two-sided arrows indicate distances between the upper or lower LBD lobes within the LBD dimer. Note, the behavior of LBD dimers during desensitization is similar between different types of AMPARs. **g-i**, Time constants of desensitization (τ_{Des}, **g**) and recovery from desensitization

(τ_{RecDes}, **h**)[27,28,43,76], and the fraction of non-desensitized channels (I_{SS}/I_{Max}, **i**) for GluA1/A2 core (this study), GluA1/A2-γ8 (full-length rat GluA1 flip, full-length rat GluA2 flip, full-length rat TARP γ8; outside-out patches from *Xenopus* oocytes, 129 ± 56 ms / mean ± SD, n = 8-19 experiments)[38], A2-γ2 (GT linker between rat $GluA2_{flip}$ and mouse γ2, truncation after L207 of γ2, 36 residues deleted from the C terminus of GluA2, six residues deleted from the ATD-LBD linker, knocked-out N-linked glycosylation sites N235E, N385D and N392Q, mutation R586Q)[32] 13.3 ± 1.0 ms / mean ± SEM, n = 4)[28], A2-GSG1L (GT linker between rat GluA2 flip crystallization construct[32] and mouse GSG1L (CTD truncation after F237); 164 ms ± 11 ms SEM, n = 7)[42], A2-γ5 (GT linker between rat GluA2 flip (crystallization construct)[32] and mouse TARP-γ5 (CTD truncation after E207); 29.8 ± 1.9 ms SEM, n = 6)[42], and A2-γ5-CNIH (36.7 ms ± 2.7 ms SEM, n = 8)[43]. of AMPARs in the absence or presence of auxiliary subunits (GluA1/A2 core (this study), GluA1/A2-γ8[76], A2-γ2[28], A2-GSG1L[42], A2-γ5[42], and A2-γ5-CNIH2[43]). **j-k**, LBD dimer D1 lobe separation (**j**) and subunits B/D D2 lobe separation (**k**) in the closed (blue circles) and open (red circles) state structures of GluA1/A2 and desensitized (green circles) state structures of GluA1/A2, GluA1/A2-γ8 (PDB ID: 7QHH), GluA2-γ2 (PDB ID: 9MRL), GluA2-GSG1L (PDB ID: 7RZA), GluA2-γ5 (PDB ID: 7RZ7) and GluA2-γ5-CNIH2 (PDB ID: 8SSB).

Supplementary Table 1. Cryo-EM data collection, refinement, and validation statistics.

Structure	GluA1/A2$_{ZK}$ LBD-TMD composite	GluA1/A2$_{ZK}$ consensus	GluA1/A2$_{ZK}$ LBD	GluA1/A2$_{ZK}$ TMD	GluA1/A2$_{Glu+RR2b}$ FL composite	GluA1/A2$_{Glu+RR2b}$ FL consensus
EMDB code	EMD-70912	EMD-70909	EMD-70911	EMD-70910	EMD-70919	EMD-70913
PDB code	9OVT	-	-	-	9OVU	-
**Data collection and processing**						
Voltage (kV)	300	300	300	300	300	300
Electron exposure ($e^{-}Å^{-2}$)	45.72	45.72	45.72	45.72	47.03	47.03
Reported pixel size (Å)	0.826	0.826	0.826	0.826	0.825	0.825
Exposure number	14,743	14,743	14,743	14,743	20,912	20,912
**Processing software**						
Particle picking	cryoSPARC-v4.1	cryoSPARC-v4.1	cryoSPARC-v4.1	cryoSPARC-v4.1	cryoSPARC-v4.1	cryoSPARC-v4.1
Motion correction	MotionCor2	MotionCor2	MotionCor2	MotionCor2	MotionCor2	MotionCor2
CTF estimation	Patch CTF	Patch CTF	Patch CTF	Patch CTF	Patch CTF	Patch CTF
2D/3D classification, refinement	cryoSPARC-v4.1	cryoSPARC-v4.1	cryoSPARC-v4.1	cryoSPARC-v4.1	cryoSPARC-v4.1	cryoSPARC-v4.1
Symmetry imposed	C2	C2	C1	C1	C2	C2
Initial particle number	4,489,530	4,489,530	4,489,530	4,489,530	5,802,290	5,802,290
Final particle number	219,104	219,104	438,208	438,208	74,183	74,183
Map resolution, FSC cut-off 0.143 (Å)	3.34	3.43	3.34	3.34	3.65	3.20
Map resolution, FSC cut-off 0.5 (Å)	3.76	3.75	3.7	3.79	4.10	3.49
**Refinement**						
FSC threshold	0.143	0.143	0.143	0.143	0.143	0.143
Map sharpening B factor ($Å^2$)	66.9	66.9	94.1	76.8	90.6	90.6
**Model composition**						
Non-hydrogen atoms	13,003	-	-	-	25,011	-
Protein residues	1,648	-	-	-	3,114	-
**Ligands**						
ZK200775	4	-	-	-	-	-
(R,R)-2b	-	-	-	-	2	-
Glutamate	-	-	-	-	4	-
NA	3	-	-	-	1	-
**B factors ($Å^2$)**						
Protein (mean)	72.43	-	-	-	160.26	-
Ligands (mean)	39.64	-	-	-	108.73	-
**R.m.s. deviations**						
Bond lengths (Å)	0.008	-	-	-	0.006	-
Bond angles (°)	1.272	-	-	-	1.248	-
**Validation**						
MolProbity score	1.69	-	-	-	1.81	-
Clash score,	3.72	-	-	-	3.88	-
Poor rotamers (%)	0.29	-	-	-	0.60	-
**Ramachandran plot**						
Favored (%)	90.75	-	-	-	86.26	
Allowed (%)	9.13	-	-	-	13.35	
Disallowed (%)	0.12	-	-	-	0.39	

Structure	GluA1/A2$_{Glu+RR2b}$ ATD	GluA1/A2$_{Glu+RR2b}$ LBD	GluA1/A2$_{Glu+RR2b}$ TMD	GluA1/A2-CNIH1$_{Glu+RR2b}$ LBD-TMD composite	GluA1/A2-CNIH1$_{Glu+RR2b}$ consensus	GluA1/A2-CNIH1$_{Glu+RR2b}$ LBD
EMDB code	EMD-75274	EMD-70914	EMD-70915	EMD-70920	EMD-70916	EMD-70917
PDB code	-	-	-	9OVV	-	-
**Data collection and processing**						
Voltage (kV)	300	300	300	300	300	300
Electron exposure ($e^{-}Å^{-2}$)	47.03	47.03	47.03	45.72	45.72	45.72
Reported pixel size (Å)	0.825	0.825	0.825	0.826	0.826	0.826
Exposure number	20,912	20,912	20,912	14,743	14,743	14,743
**Processing software**						
Particle picking	cryoSPARC-v4.1	cryoSPARC-v4.1	cryoSPARC-v4.1	cryoSPARC-v4.1	cryoSPARC-v4.1	cryoSPARC-v4.1
Motion correction	MotionCor2	MotionCor2	MotionCor2	MotionCor2	MotionCor2	MotionCor2
CTF estimation	Patch CTF	Patch CTF	Patch CTF	Patch CTF	Patch CTF	Patch CTF
2D/3D classification, refinement	cryoSPARC-v4.1	cryoSPARC-v4.1	cryoSPARC-v4.1	cryoSPARC-v4.1	cryoSPARC-v4.1	cryoSPARC-v4.1
Symmetry imposed	C1	C1	C2	C2	C2	C1
Initial particle number	5,802,290	5,802,290	5,802,290	4,489,530	4,489,530	4,489,530
Final particle number	121,113	151,693	61,870	76,183	76,183	56,116
Map resolution, FSC cut-off 0.143 (Å)	3.94	3.1	3.9	3.76	3.76	3.51
Map resolution, FSC cut-off 0.5 (Å)	4.44	3.5	4.3	4.06	3.91	4.0
**Refinement**						
FSC threshold	0.143	0.143	0.143	0.143	0.143	0.143
Map sharpening B factor ($Å^2$)	141.0	96.0	128.9	105.0	105.0	89.0
**Model composition**						
Non-hydrogen atoms	-	-	-	17,757	-	-
Protein residues	-	-	-	2,106	-	-
**Ligands**						
(R,R)-2b	-	-	-	2	-	-
Glutamate	-	-	-	-	4	-
NA	-	-	-	1	-	-
POV	-	-	-	16	-	-
**B factors ($Å^2$)**						
Protein (mean)	-	-	-	71.12	-	-
Ligands (mean)	-	-	-	33.36	-	-
**R.m.s. deviations**						
Bond lengths (Å)	-	-	-	0.009	-	-
Bond angles (°)	-	-	-	1.441	-	-
**Validation**						
MolProbity score	-	-	-	1.67	-	-
Clash score	-	-	-	4.02	-	-
Poor rotamers (%)	-	-	-	0.22	-	-
**Ramachandran plot**						
Favored (%)	-	-	-	92.00	-	-
Allowed (%)	-	-	-	7.80	-	-
Disallowed (%)	-	-	-	-	-	-

Structure	GluA1/A2-CNIH1$_{Glu+RR2b}$ TMD	GluA1/A2$_{Quis}$ FL composite	GluA1/A2$_{Quis}$ consensus	GluA1/A2$_{Quis}$ ATD	GluA1/A2$_{Quis}$ LBD	GluA1/A2$_{Quis}$ TMD
EMDB code	EMD-70918	EMD-70925	EMD-70921	EMD-70922	EMD-70923	EMD-70924
PDB code	-	9OVW	-	-	-	-
**Data collection and processing**						
Voltage (kV)	300	300	300	300	300	300
Electron exposure ($e^{-}Å^{-2}$)	45.72	45.8	45.8	45.8	45.8	45.8
Reported pixel size (Å)	0.826	1.35	1.35	1.35	1.35	1.35
Exposure number	14,743	13,380	13,380	13,380	13,380	13,380
**Processing software**						
Particle picking	cryoSPARC-v4.1	cryoSPARC-v4.1	cryoSPARC-v4.1	cryoSPARC-v4.1	cryoSPARC-v4.1	cryoSPARC-v4.1
Motion correction	MotionCor2	MotionCor2	MotionCor2	MotionCor2	MotionCor2	MotionCor2
CTF estimation	Patch CTF	Patch CTF	Patch CTF	Patch CTF	Patch CTF	Patch CTF
2D/3D classification, refinement	cryoSPARC-v4.1	cryoSPARC-v4.1	cryoSPARC-v4.1	cryoSPARC-v4.1	cryoSPARC-v4.1	cryoSPARC-v4.1
Symmetry imposed	C2	C2	C2	C1	C2	C2
Initial particle number	4,489,530	1,154,987	1,154,987	1,154,987	1,154,987	1,154,987
Final particle number	76,183	251,523	251,523	87,480	89,333	32,669
Map resolution, FSC cut-off 0.143 (Å)	3.55	4.48	4.91	4.28	4.59	4.57
Map resolution, FSC cut-off 0.5 (Å)	4.11	6.03	6.49	5.11	6.14	6.83
**Refinement**						
FSC threshold	0.143	0.143	0.143	0.143	0.143	0.143
Map sharpening B factor ($Å^2$)	133.4	293.1	293.1	170.3	259.1	197.5
**Model composition**						
Non-hydrogen atoms	-	25,104	-	-	-	-
Protein residues	-	3,154	-	-	-	-
**Ligands**						
Quisqualate	-	4	-	-	-	-
NAG	-	6	-	-	-	-
**B factors ($Å^2$)**						
Protein (mean)	-	149.15	-	-	-	-
Ligands (mean)	-	156.84	-	-	-	-
**R.m.s. deviations**						
Bond lengths (Å)	-	0.006	-	-	-	-
Bond angles (°)	-	1.308	-	-	-	-
**Validation**						
MolProbity score	-	2.20	-	-	-	-
Clash score	-	6.02	-	-	-	-
Poor rotamers (%)	-	0.23	-	-	-	-
**Ramachandran plot**						
Favored (%)	-	81.13	-	-	-	-
Allowed (%)	-	18.36	-	-	-	-
Disallowed (%)	-	0.51	-	-	-	-
