# Supplementary material for: Auxiliary subunits reshape structural asymmetry and functional plasticity in heterotetrameric GluA1/A2 AMPA receptor core
Source: Nat Commun. 2026 Mar 28;17:4191. doi: 10.1038/s41467-026-71063-1 (PMC13153358; doi:10.1038/s41467-026-71063-1)
Supplement: Supplementary file 2 — Description of Additional Supplementary Files [file 41467_2026_71063_MOESM2_ESM.pdf]

Description of Additional Supplementary Files

File Name: Supplementary Data 1

Description: Mass spectrometry (MS) analysis of the purified protein sample to identify which specific paralogs of endogenous human CNIHs, CNIH1-4, can be represented by the cryo-EM density.
